# Reduced interfacial recombination in dye-sensitized solar cells assisted with NiO:Eu^3+^,Tb^3+^ coated TiO_2_ film

**DOI:** 10.1038/srep31123

**Published:** 2016-08-10

**Authors:** Nannan Yao, Jinzhao Huang, Ke Fu, Xiaolong Deng, Meng Ding, Shouwei Zhang, Xijin Xu, Lin Li

**Affiliations:** 1 School of Physics and Technology, University of Jinan, Jinan 250022, Shandong Province, P. R. China; 2 Key Laboratory for Photonic and Electric Bandgap Materials, Ministry of Education, Harbin Normal University, Harbin, 150025, Heilongjiang Province, P. R. China

## Abstract

Eu^3+^,Tb^3+^ doped and undoped NiO films were deposited on TiO_2_ by a sol-gel spin-coating method as the photoanodes of dye sensitized solar cells (DSSCs). A comparative study with different structures including TiO_2_, TiO_2_/NiO and TiO_2_/NiO:Eu^3+^,Tb^3+^ as the photoanodes was carried out to illustrate the photovoltaic performance of solar cells. NiO could enhance the performance of DSSCs ascribed to acting as a barrier for the charge recombination from the fluorine doped tin oxide (FTO) to electrolyte and forming a p-n junction (NiO/TiO_2_). Moreover, Eu^3+^, Tb^3+^ co-doped NiO could accelerate the electron transfer at TiO_2_/dye/electrolyte interface, which further benefited the performance of solar cells. The solar cells assembled with the photoelectrodes consisting of NiO:Eu^3+^,Tb^3+^ and TiO_2_ exhibited short-circuit current density (*J*_*SC*_) of 17.4 mA cm^−2^, open-circuit voltage (*V*_*OC*_) of 780 mV and conversion efficiency of 8.8%, which were higher than that with TiO_2_/NiO and pure TiO_2_. The mechanisms of the influence of NiO and NiO:Eu^3+^,Tb^3+^ on the photovoltaic performance of DSSCs were discussed.

Since O’Regan and Grätzel reported their breakthrough results in 1991, dye sensitized solar cells (DSSCs) as the next generation of solar cells have received tremendous interests due to their low cost and high theoretical performance^1^,^2^. In recent years, many efforts have been made to improve the performance of solar cells and an efficiency of 14.5% was reached for the liquid-based DSSCs^3^. A typical DSSC is composed of a dye sensitized nanocrystalline TiO_2_ film, an electrolyte containing I_3_^−^/I^−^ redox couple, and a Pt counter electrode^4^. TiO_2_ photoanode structures play a crucial role in the charge transport and the amount of adsorbed dye molecules, which would significantly affect the overall energy conversion^5^,^6^. Therefore, intensive research has focused on the modification of working electrode with high surface area, fast electron transport pathways, and so on, which would remarkably improve the performance of DSSCs^7^,^8^,^9^.

In this context, many cited studies are concerned with the engineering of the photoanode interfaces in DSSCs^10^,^11^,^12^. As is well known, several important processes occur at TiO_2_/dye/electrolyte interface, such as the electrons injection from dyes to the conduction band of TiO_2_ and the back reaction of injected electron in nanocrystalline films with electrolyte and oxidized dyes^13^,^14^. In order to facilitate electron transport and reduce the charge recombination, several research groups have recently attempted to modify the surface of TiO_2_ with a thin insulating layer by forming an energy barrier between TiO_2_ electrodes and electrolyte^15^,^16^. For example, Palomares *et al*. have grown the insulating layer on nanocrystalline TiO_2_ semiconductor films to modulate the interfacial electron transfer in DSSCs, and the cells with a Al_2_O_3_ overlayer achieved an enhancement in *I*_*SC*_ and *V*_*OC*_^17^. Many metal oxides with wide bandgap like MgO^18^, NiO^19^ and Nb_2_O_5_^20^ have been developed to deposit on TiO_2_ film as surface passivation for the purpose of accelerating the electron transfer at interface. Among these metal oxide materials, p-type semiconductor NiO with a wide band gap (Eg = 3.55 eV) shows excellently thermal and chemical stability, which has become a promising material in photoelectrochemical devices^21^. The combination of p-NiO and n-TiO_2_ has been proven to facilitate charge separation, which in turn leads to the enhancement of the V_OC_ and the conversion efficiency in DSSCs^22^. Moreover, the modification of NiO by doping with impurities such as various metal ions has been demonstrated to be an efficient approach to improve the photoelectrochemical properties^23^. For example, lithium as dopant to increase the carrier concentration and mobility and improve the electrical conductivity of NiO, which has been reported to be potential material for applications at gas sensor and p-type DSSCs^24^,^25^.

In this study, the composite electrodes comprising n-type TiO_2_ and p-type NiO:Eu^3+^,Tb^3+^ sensitized with N719 are for the first time applied to construct DSSCs. For better confirming the effect of NiO and NiO:Eu^3+^,Tb^3+^ on the performance of solar cells, the comparisons between the DSSC with the above composite working electrode and the conventional TiO_2_-based DSSC were also made in this paper.

## Methods

### Synthesis of NiO and NiO:Eu^3+^,Tb^3+^

NiO and NiO:Eu^3+^,Tb^3+^ were prepared using a sol-gel method. Typically, 0.05 M nickel acetate was dissolved into a mixing solution of 2-Methoxyethanol and Monoethanolamine under stirring at 60 °C, and the molar ratio of nickel acetate to Monoethanolamine is 1:1. Moreover, in order to synthesis NiO:Eu^3+^,Tb^3+^, Eu(NO_3_)_3_·6H_2_O and Tb(NO_3_)_3_·6H_2_O were dissolved into the above solution with a molar ratio of NiO to RE^3+^ being 1:0.01:0.01 under stirring. Then, NiO and NiO:Eu^3+^,Tb^3+^ sol were obtained after stirring for 1 h. In order to collect the sample powers for further analysis, the obtained sol was dried at 80 °C, followed by annealing at 500 °C for 2 h.

### DSSCs assembly

Prior to the fabrication of DSSCs, fluorine doped tin oxide (FTO, transmittance = 85%) conductive glasses acted as substrates were cleaned with acetone, ethanol and deionized (DI) water, followed by drying. TiO_2_ sol prepared as the previous method was spin-coated on FTO to suppress the back electron transfer from FTO to electrolyte^26^. To prepare porous TiO_2_ working electrodes, the pastes were coated on substrates by the doctor blade method, followed by sintering at 450 °C for 30 min. After cooling down to room temperature, NiO and NiO:Eu^3+^,Tb^3+^ layer were deposited on the bare TiO_2_ electrode to form TiO_2_/NiO and TiO_2_/NiO:Eu^3+^,Tb^3+^ composite structures by spin-coating at 3000 rpm for 30s. After annealing at 500 °C for 2 h, these samples were immersed into N719 ethanol solution for 24 h. Pt (OPV-Pt-S) counter electrode was spin-coated on FTO glass and annealed at 450 °C for 30 minutes. Subsequently, the dye-sensitized photoanodes and Pt counter electrode were fixed together using a hot-melt film spacer. Finally, a DSSC was assembled by injecting electrolyte (OPV-MPN-I) into the space between the electrodes.

### Characterization and measurement

The phase structure of NiO and NiO:Eu^3+^,Tb^3+^ was characterized by X-ray diffraction (XRD) using a D8 ADVANCE with Cu *K*_α_ at λ = 0.15406 nm. X-ray photo-electron spectroscopy (XPS) was performed on a Thermo ESCALAB 250XI electron spectrometer equipped with Al Ka X-ray radiation (E = 1486.6 eV) as the source for excitation. The photoluminescence (PL) of samples was measured with Fluoromax-4 spectrometer made by HORIBA. The surface and cross-section morphology of photoanode structures were observed by field emission scanning electron microscope (FE-SEM, Quanta FEG250). The optical absorption spectra of different composite anode films and dye desorbed from photoanodes were recorded by UV-vis-NIR spectrophotometer (TU-1901). The electrochemical measurements were performed by an electrochemical analyzer in a standard three-electrode system with composite films coated-FTO as the working electrode, platinum wire as the counter electrode, Ag/AgCl electrodes as the reference electrode, and the supporting electrolyte was 0.5 M Na_2_SO_4_ aqueous solution. *I*–*V* characteristics of the DSSCs were measured with an Aglient B2901A source/meter under a Xe lamp. The irradiation areas of the working electrode were 0.16 cm^2^. All of these measurements were carried out at room temperature.

## Results and Discussion

**Figure 1:**
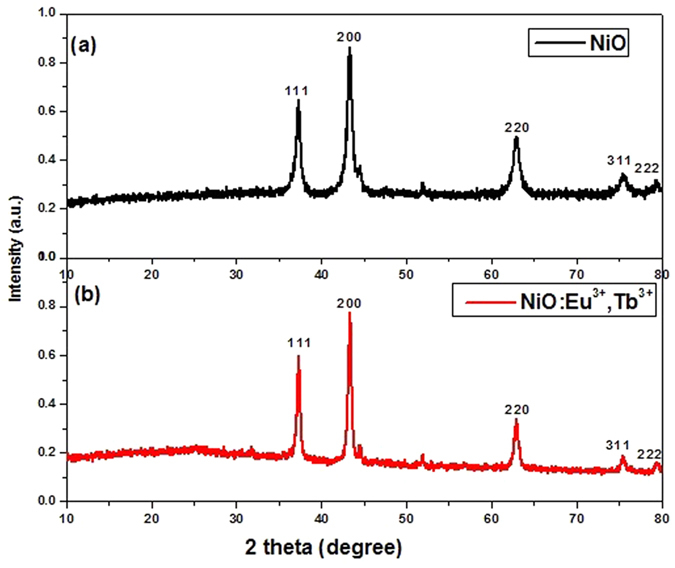
XRD patterns of (a) NiO and (b) NiO:Eu^3+^,Tb^3+^.

The XRD patterns are performed to characterize the influence of rare earth ions dopant on the crystallization of NiO, as shown in Fig. 1. The observed diffraction peaks (111), (200), (220), (311) and (222) can be readily indexed to the cubic phase of NiO (JCPDS No. 44–1159). No other impurity diffraction peaks are observed, indicating that rare earth ions might incorporate into the lattice. The sharp peaks observed from XRD patterns confirm the formation of highly crystalline NiO phase. The crystallite size of the nanocrystals was calculated about 10 nm by Scherrer formula. Apparently, the crystallite size *D* correlated inversely with full-width half-maximum of the diffraction peak *β*. Thus, the size of NiO:Eu^3+^,Tb^3+^ is slightly larger than pure NiO due to the smaller *β*. Indeed, the incorporation of rare earth ions may lead to lattice distortion, which can be reasonably explained by the fact that the ionic radii of Eu^3+^ and Tb^3+^ is larger than that of Ni^2+^.

**Figure 2:**
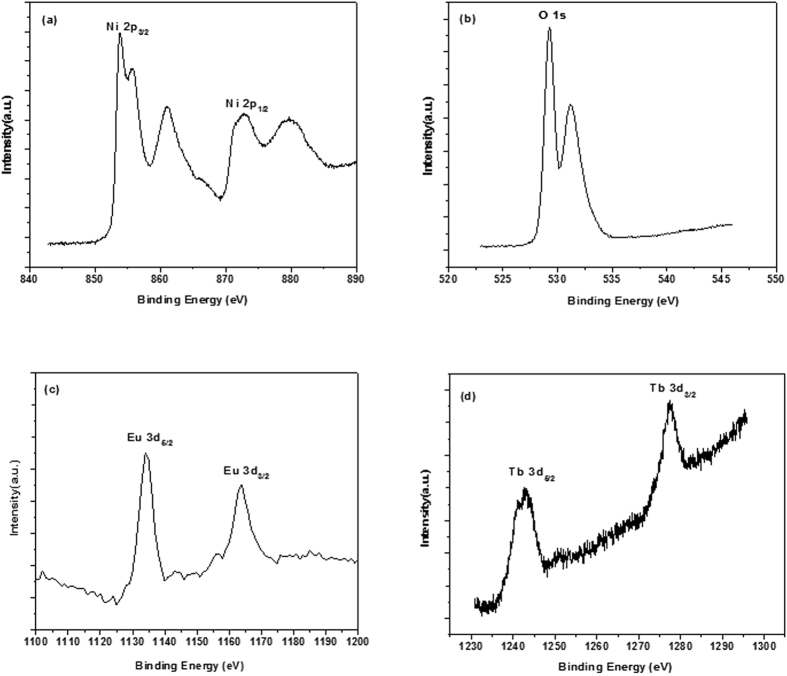
XPS spectra of (a) Ni 2p, (b) O 1s, (c) Eu 3d and (d) Tb 3d for NiO:Eu^3+^,Tb^3+^.

In order to characterize the doping of Eu^3+^,Tb^3+^, the XPS measurement was conducted as shown in Fig. 2. As presented in Fig. 2(a), the Ni 2p region comprises four peaks: the main peak in Ni 2p_3/2_ was located at 854 eV while its satellite was detected at 862 eV, and the peaks at 872 and 879 eV corresponded to Ni 2p_1/2_ main peak and its satellite respectively. And Fig. 2(b) shows two peaks resulting from the lattice oxygen at 529 eV and 531 eV. The Eu 3d_5/2_ and Eu 3d_3/2_ (Fig. 2(c)) binding energy peak positions were found at 1135 eV and 1165 eV, while the broad peak at 1242 eV and 1280 eV were identified as Tb 3d (Fig. 2(d)), suggesting the presence of Eu and Tb in the sample. However, the diffraction peaks related to Eu and Tb were not observed in Fig. 1(b), it was certain that Eu and Tb ions had been successfully incorporated in NiO lattice.

**Figure 3:**
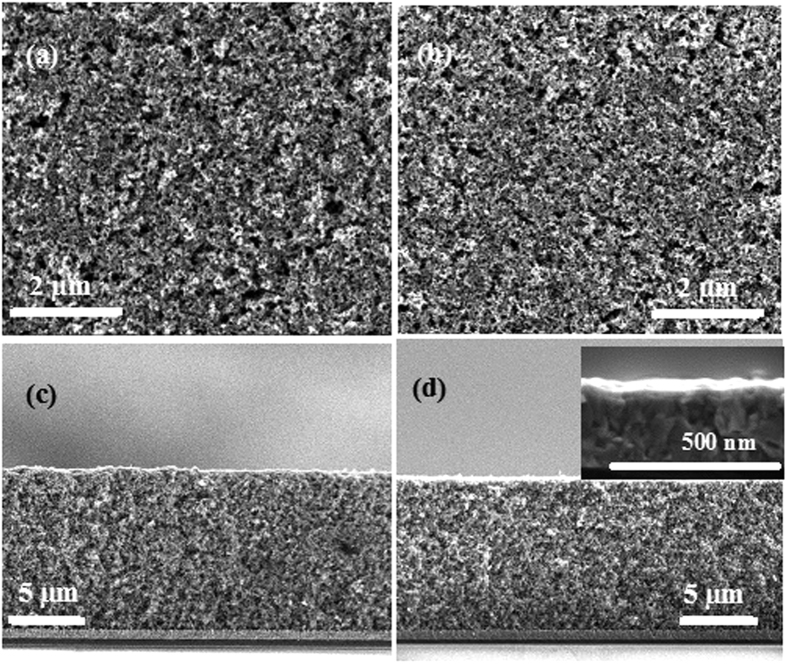
Surface and cross-section SEM images of FTO/TiO_2_ (a,c) and FTO/TiO_2_/NiO (b,d). The inserts show cross-section image of NiO coated on FTO.

Figure 3 shows the surface and cross-section SEM images of the as-prepared photoanodes FTO/TiO_2_/NiO and FTO/TiO_2_. From the top view observation of the photoanode, both samples exhibit the uniform surface morphologies with high porosity. The thickness of the composite films is about 12 μm as shown in the cross-sectional SEM images (Fig. 3(c,d)), and a very thin NiO layer with average thickness of 50 nm directly coated on FTO was observed in the inset.

**Figure 4:**
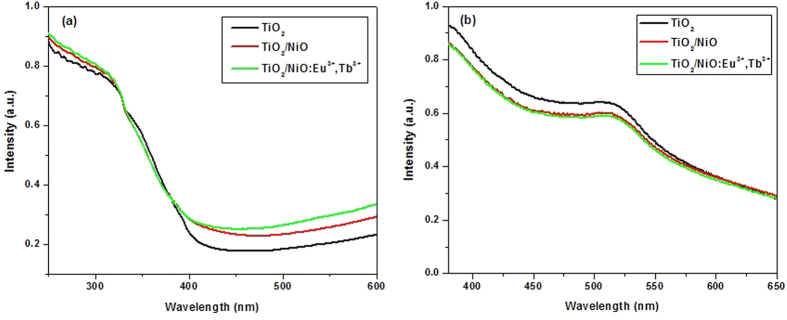
Absorption spectra of (a) TiO_2_, TiO_2_/NiO, TiO_2_/NiO:Eu^3+^,Tb^3+^ and (b) dye desorbed from different photoanodes.

The absorption spectra of TiO_2_, TiO_2_/NiO, TiO_2_/NiO:Eu^3+^,Tb^3+^ and dye desorbed from various photoanodes were measured. From Fig. 4(a), it can be seen that the absorption intensity of TiO_2_/NiO and TiO_2_/NiO:Eu^3+^,Tb^3+^ is higher than that of pure TiO_2_, which can be ascribed to the UV-light absorption of rare earth ions and the slightly increasing thickness of composite films. Furthermore, the presence of doped Eu^3+^ and Tb^3+^ can create an impurity energy level, causing the absorption spectra shifted to lower energy region. Therefore, the absorption could be significantly enhanced in 400 nm–600 nm region as shown in Fig. 4(a), extending the photoresponse to visible light for DSSCs. In order to measure the dye loading amount of different samples, the absorption spectra of dye desorbed from various photoanodes in NaOH solution was investigated according to the previous literature^7^. Typically, the different photoanodes sensitized by dye were immersed in NaOH solution for several minutes, then the N719 can be desorbed from photoanode and dissolved into the NaOH solution. As can be seen in Fig. 4(b), the absorption spectra of dye desorbed from TiO_2_/NiO and TiO_2_/NiO:Eu^3+^,Tb^3+^ films was similarly, which were all lower than raw TiO_2_ film due to the reduced surface area of photoanodes.

**Figure 5:**
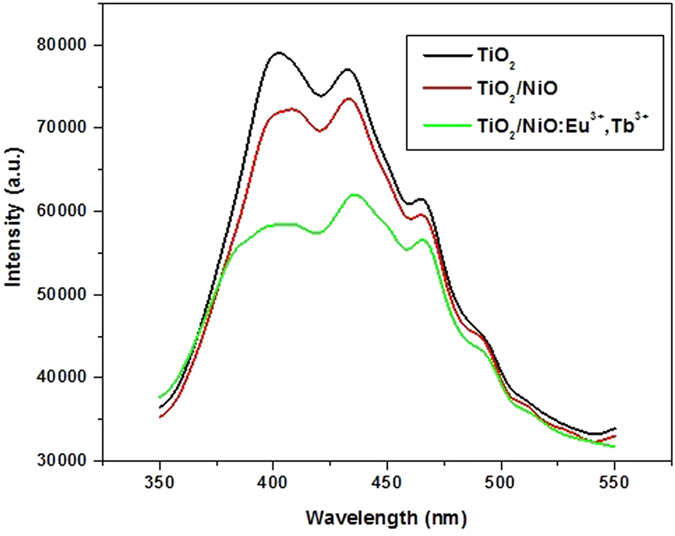
PL spectra of TiO_2_, TiO_2_/NiO and TiO_2_/NiO:Eu^3+^,Tb^3+^.

To study the separation efficiency of photogenerated electrons and holes, the room temperature PL spectra of the as-synthesized composite structure TiO_2_, TiO_2_/NiO and TiO_2_/NiO:Eu^3+^,Tb^3+^ were carried out, respectively. Figure 5 exhibits the PL spectra of all samples excited at 300 nm. It is obvious that the PL intensity follows the sequence of TiO_2_>TiO_2_/NiO>TiO_2_/NiO:Eu^3+^,Tb^3+^. The decrease of PL intensity indicates the efficient electron-hole separation and long-lived carriers. In this study, depositing a thin NiO layer on TiO_2_ could form a p-n junction, which facilitates the charge separation. In addition, rare earth ions could enter into the lattice of NiO and produce the shallow trapper, promoting the lifetime of carrier and reducing the recombination of electrons and holes effectively. Therefore, it is conclusion that the presence of NiO:Eu^3+^,Tb^3+^ could be effective to enhance the photovoltaic performance of DSSCs.

**Figure 6:**
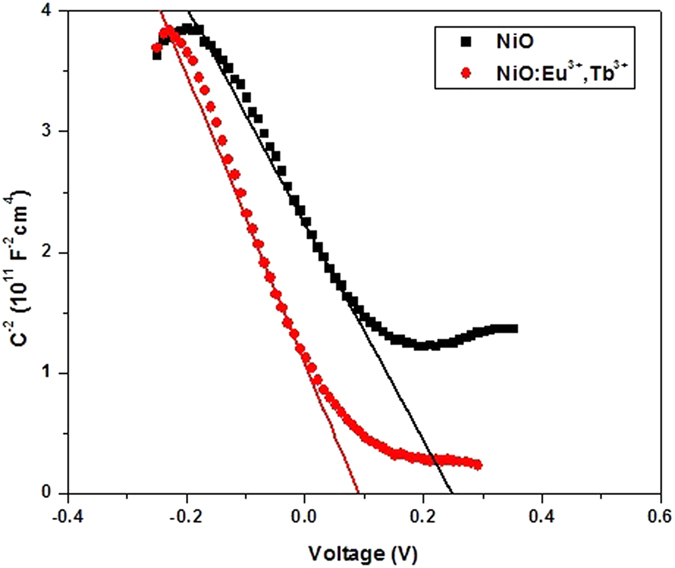
MS plots of (a) NiO and (b) NiO:Eu^3+^,Tb^3+^ in 0.5 M Na_2_SO_4_ electrolyte.

The effect of dopants on NiO was investigated and discussed. Firstly, according to the results of XRD and XPS measurements for NiO:Eu^3+^,Tb^3+^, it was certain that Eu and Tb ions had been successfully incorporated in NiO. The Mott-Schottky (MS) measurement for NiO and NiO:Eu^3+^,Tb^3+^ film was also conducted. As shown in Fig. 6, the curves show negative slopes, consisting with p-type semiconductor. According to the equation *1*/*C*^*2*^ = (*2/qεε_0_N_D_*)(*E − E_FB_ − kT/q*), where C is capacitance of the space charge region, *ε* is dielectric constant of the semiconductor, *ε*_*0*_ is permittivity of free space, N_D_ is donor density, E is applied potential, and E_FB_ is flatband potential. The charge carrier concentration N_D_ is negative correlated with the slopes, and the smaller slope of NiO:Eu^3+^,Tb^3+^ corresponds to higher carrier concentration than NiO. The increased carrier concentration of Eu^3+^,Tb^3+^ doped NiO will exhibit better electrical conductivity, leading to easier electron transfer from dye to TiO_2_ and excellent performance of DSSCs.

**Figure 7:**
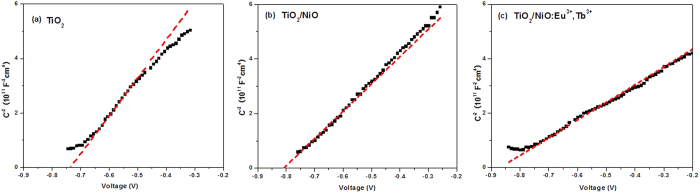
MS plots of (a) TiO_2_, (b) TiO_2_/NiO and (c) TiO_2_/NiO:Eu^3+^,Tb^3+^ in 0.5 M Na_2_SO_4_ electrolyte.

To understand the reasons caused an enhancement of charge separation efficiency for the solar cell with a thin barrier layer, the flat band potential E_FB_ of different three composite films were also evaluated by MS measurement. Figure 7 displays the MS plots of TiO_2_, TiO_2_/NiO and TiO_2_/NiO:Eu^3+^,Tb^3+^ respectively, and all the samples show positive slopes, consisting with the expected n-type semiconductor characteristics. According to the above equation *1*/*C*^*2*^ = (*2/qεε_0_N_D_*)(*E − E_FB_ − kT/q*), *E*_*FB*_ is determined by extrapolating 1/C^2^ to 0. It is clearly observed the negative shift of flat-band of TiO_2_/NiO:Eu^3+^,Tb^3+^ compared to that of TiO_2_ and TiO_2_/NiO in Fig. 6, which suggests the increase of electron concentration and more positive Fermi levels *E*_*F*_. In addition, the NiO:Eu^3+^,Tb^3+^-coated TiO_2_ layer exhibits a smaller slope than other samples, indicating an increased carrier densities in TiO_2_, thus the *J*_*SC*_ values of solar cells could be probably increased. Moreover, the generated voltage in DSSCs corresponds to the difference between the Fermi level of the mesoporous semiconductor and the redox potential of the electrolyte^27^. Therefore, the energy gap between TiO_2_ Fermi level and redox potential will be enlarged with the elevated *E*_*F*_, contributing to a higher *V*_*OC*_ value in DSSCs.

**Figure 8:**
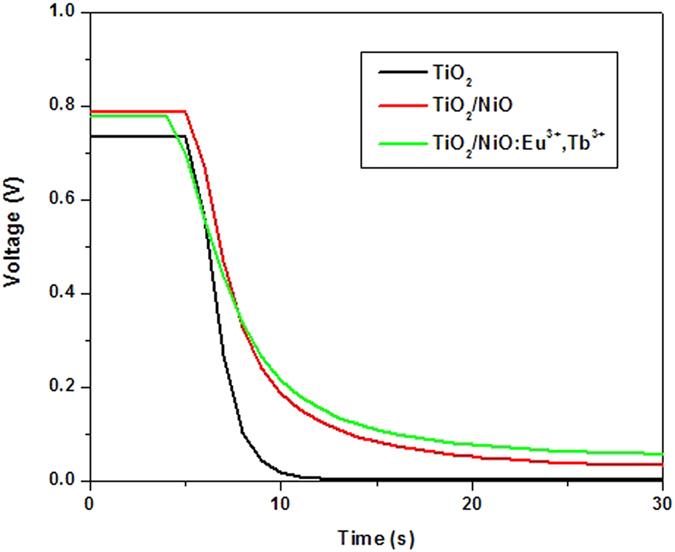
Decay results of V_OC_ for the bare and barrier-layer coated DSSCs.

The electron collection efficiency and charge recombination at TiO_2_/electrolyte interface was analyzed by the open-circuit voltage decay, which monitors the photovoltage decay of devices through interrupting a steady-state illumination^28^. Under constant illumination, the free electron concentration *n* increases with the concentration *n*_*0*_ in the dark. The open circuit voltage of solar cell is determined by the equation *V_OC_* = (*E_F_* − *E_0_*)/*e* = *K_B_T/e ln*(*n/n_o_*). thus there was a positive correlation between *Voc* and electron concentration *n*. As shown in Fig. 8, the photovoltage keeps a constant value under illumination with the order of TiO_2_/NiO:Eu^3+^,Tb^3+^>TiO_2_/NiO>TiO_2_ for DSSCs, suggesting an increased free electron concentration in TiO_2_/NiO:Eu^3+^,Tb^3+^ and TiO_2_/NiO composite structures. A subsequent decay process of voltage occurs via interrupting the steady-state illumination, in which the free electron density in the semiconductor transforms from the initial steady state to the dark equilibrium, indicating the recombination process of electrons in the conduction band of TiO_2_ with the electrolyte. The slow decay responses of TiO_2_/NiO and TiO_2_/NiO:Eu^3+^,Tb^3+^ composite structures indicates that the introduction of NiO and NiO:Eu^3+^,Tb^3+^ could decrease the interfacial charge recombination compared to the pure TiO_2_ film. In addition, the electron lifetime is given by the equation *τ* = −*K_B_T/e (dV_OC_/dt*)^−1^. thus the smaller slope during the decay process indicating the longer electron lifetime. The longer lifetime for TiO_2_/NiO:Eu^3+^,Tb^3+^ based DSSCs is due to the presence of barrier layer, which could reduce the back-transfer of electron from TiO_2_ to electrolyte.

**Figure 9:**
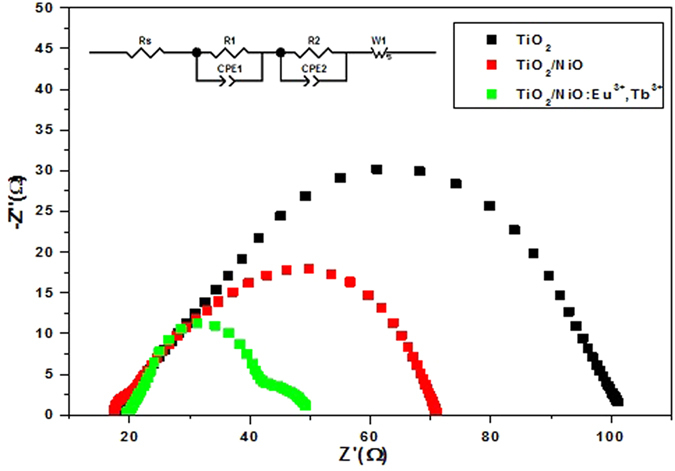
Impedance spectra of the devices with TiO_2_, TiO_2_/NiO and TiO_2_/NiO:Eu^3+^,Tb^3+^ under illumination.

Electrochemical impedance spectroscopy (EIS) technique had been widely employed to investigate internal resistance and charge transfer process in DSSCs^29^. As shown in Fig. 9, the Nyquist diagrams of solar cells with three different photoelectrodes under illumination exhibit two semicircles, corresponding to the electron transfer at the dye-sensitized photoelectrode/electrolyte interface and Pt/electrolyte interface resistance, respectively. The larger the radius of the middle frequency semicircle is in the Nyquist plots, the higher charge transfer resistance at TiO_2_/dye/electrolyte achieves. Apparently, in middle frequency, bare TiO_2_-based solar cell possesses a larger radius than the devices assembled with TiO_2_ film coated by NiO and NiO: Eu^3+^,Tb^3+^. The decrease in the radius reveals the lower interface resistance, which is mainly attributed to the reduction of direct exposure area of TiO_2_ to the electrolyte with the presence of passivation layer. Meanwhile, the conductivity of semiconductor was improved by doping RE^3+^ ions, resulting in accelerating electron transfer at the interface. Therefore, the solar cells assembled with NiO: Eu^3+^,Tb^3+^ exhibit lower interface resistance, so as to achieve a faster interfacial electron transport and higher photoelectric performance.

**Figure 10:**
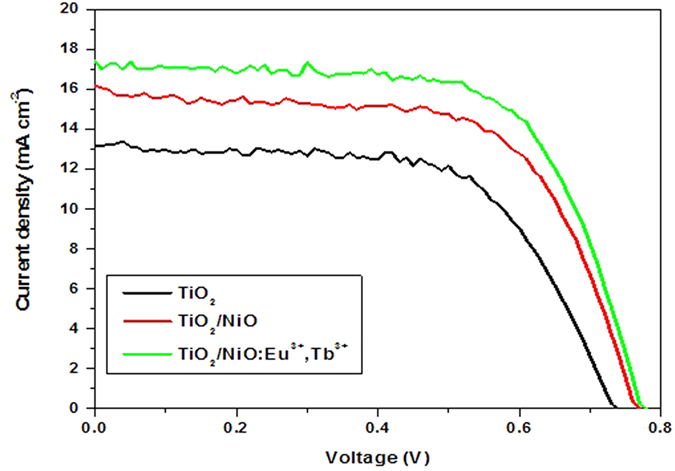
I-V curves of the DSSCs made from TiO_2_, TiO_2_/NiO and NiO:Eu^3+^,Tb^3+^ electrodes.

The influence of NiO and NiO:Eu^3+^,Tb^3+^ on *I-V* characteristics of DSSCs was also investigated. Figure 10 shows *I-V* curves of the DSSCs based on TiO_2_, TiO_2_/NiO and TiO_2_/NiO:Eu^3+^,Tb^3+^ electrodes under illumination respectively, and Table 1 presents corresponding photovoltaic parameters of solar cells. The typical DSSC using pure TiO_2_ shows short circuit current density *(J*_*SC*_) of 13.15 mA cm^−2^, open circuit voltage (*V*_*OC*_) of 0.74 V, fill factor (*FF*) of 0.63, and power conversion efficiency (PCE) of 6.17%, which are lower than that of devices with NiO or NiO:Eu^3+^,Tb^3+^ layer. The charge recombination between TiO_2_ and electrolyte leads to the decreased performance of solar cells to a large extent. When depositing a thin NiO layer, the performance has improved remarkably, showing a *J*_*SC*_ of 16.18 mA cm^−2^, and a *V*_*OC*_ of 0.77 V, yielding 7.81% conversion efficiency. The enhancement is ascribed to the presence of barrier at the interface between TiO_2_ film and electrolyte. As the previous reports, an insulate oxide layer coated on nanoporous films acted as a barrier for charge recombination can induce the improvement in I_SC_ and *V*_*OC*_^30^. Similarly, the function of NiO could be explained act as a barrier layer for charge recombination between electrolyte and the electrons in the TiO_2_ conduction band, which can be confirmed by the results of open-circuit voltage decay. The cell efficiency could be effectively improved by retarding the recombination. Moreover, p-type semiconductor NiO integrated with n-type semiconductor TiO_2_ could form a p-n junction, which facilitates the charge separation. In addition, Eu^3+^,Tb^3+^ co-doped NiO with higher conductivity could further suppress the recombination of carriers by accelerating the interfacial electron transfer through the above analysis. It is clear that an optimal photovoltaic performance with *J*_*SC*_ = 17.4 mA cm^−2^, *V*_*OC*_  = 0.78 V, and *η* = 8.8% was achieved by the introduction of NiO:Eu^3+^,Tb^3+^ in cells. At the same time, an obvious increase in *V*_*OC*_ was obtained due to the retarded back-reaction of injected electron transfer at TiO_2_/dye/electrolyte interface.

**Figure 11:**
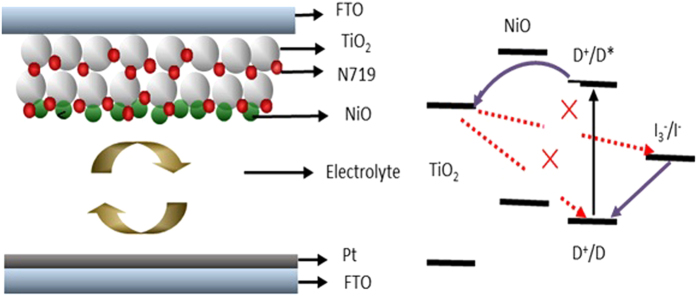
The schematic diagrams of DSSCs.

The schematic diagrams of the electron transfer path in the dye-sensitized photoanodes were depicted in Fig. 11. For the conventional DSSCs, the excited electrons were directly transferred from dye to the conduction band (CB) of TiO_2_. When depositing a thin NiO or NiO:Eu^3+^,Tb^3+^ layer on TiO_2_ nanocrystalline films, the dye can adhere to the surface of TiO_2_ or NiO particles. According to the potential level, the excited electrons of the dye adsorbed on NiO cannot be injected into the CB of NiO because the dye excited level is below than the CB potential level of NiO. However, another electron transfer path is possible that the photo-induced electrons may transfer to the CB of TiO_2_ via tunneling through NiO layer. NiO and NiO:Eu^3+^,Tb^3+^ layer could act as a barrier layer for charge recombination between the electrons in the CB of TiO_2_ and the oxided dye or electrolyte and a p-n junction was formed between TiO_2_ and NiO or NiO:Eu^3+^,Tb^3+^, which would facilitate the charge separation. Moreover, Eu^3+^,Tb^3+^ doped NiO increased carrier concentration and electrical conductivity, leading to easier electron transfer at TiO_2_/dye/electrolyte interface in DSSCs.

## Conclusions

Eu^3+^, Tb^3+^ co-doped and undoped NiO films were deposited on TiO_2_ constructing photoanode of DSSCs and an efficient enhancement in photovoltaic performance for cells was obtained. In this study, the function of a thin NiO:Eu^3+^,Tb^3+^ layer on TiO_2_ particles were summarized: (a) it acted as a barrier for the recombination of electrons in CB of TiO_2_ and the oxidized dye/electrolyte interface; (b) the n-p junction that formed between TiO_2_ and NiO could facilitate the electron transfer from NiO to TiO_2_; (c) the introduction of rare earth ions could further increase carrier concentration and improve the electron transport in solar cells. Therefore, the composite electrode TiO_2_/NiO and TiO_2_/NiO:Eu^3+^,Tb^3+^ is a promising method to modify photoanode of DSSCs with excellent solar-to-electric efficiency.

## Additional Information

**How to cite this article**: Yao, N. *et al*. Reduced interfacial recombination in dye-sensitized solar cells assisted with NiO:Eu^3+^,Tb^3+^ coated TiO_2_ film. *Sci. Rep.* **6**, 31123; doi: 10.1038/srep31123 (2016).

## Figures and Tables

**Table 1:** Photovoltaic parameters of DSSCs based on TiO_2_, TiO_2_/NiO and NiO:Eu^3+^,Tb^3+^ electrodes.

Photoanode	J_SC_(mA cm^−2^)	V_OC_(V)	FF	η(%)
TiO_2_	13.15	0.74	0.63	6.17
TiO_2_/NiO	16.18	0.77	0.63	7.81
TiO_2_/NiO: Eu^3+^,Tb^3+^	17.40	0.78	0.65	8.80
